# Factors contributing to educational differences in obesity among women: evidence from South Korea

**DOI:** 10.1186/s12889-020-09221-3

**Published:** 2020-07-20

**Authors:** Woojin Chung, Seungji Lim

**Affiliations:** 1 Department of Health Policy and Management, Graduate School of Public Health and Institute of Health Services Research, Yonsei University, 50-1 Yonsei-ro, Seodaemun-gu, Seoul, 03722 Republic of Korea; 2 Health Insurance Research Institute, National Health Insurance Service, 32, Sambo-ro, Wonju-si, Gangwon-do 26464 Republic of Korea

## Abstract

**Background:**

Obesity is more prevalent among less-educated women than highly-educated women around the world. However, little is known about the factors which cause this difference in obesity, and almost nothing is known about how the individual factors which explain differences in education among women alone contribute to obesity. In this study, we identified the factors which help explain the relationship between education and obesity in women, and quantified their separate contributions to obesity.

**Methods:**

We analyzed information on 14,577 women aged 25 years or over using datasets from the Korea National Health and Nutrition Examination Survey (2010–2014). We divided the women into two education groups: women who had, at most, finished high school (less-educated women), and women who had college degrees and beyond (highly-educated women). Using an extended Oaxaca-Blinder method, we decomposed the difference in obesity prevalence between the two education groups into the contributions (%) due to two effects: composition effect and association effect.

**Results:**

Obesity was more than twice as prevalent among the less-educated women (34.3%) than it was among the highly-educated women (16.0%). The composition effect—contribution of differences in the distribution of observed characteristics compared to that of the difference in obesity prevalence between the two education groups—was 38.2%. The association effect—contributions of differences in the estimated coefficients of characteristics compared to that of the difference in obesity prevalence between the two education groups—was 55.8%, of which lifestyle factors were the most important contributor (43.6%). Of the separate contributions of each factor, the association effect of the factor related to women’s stress exhibited the largest contribution (23.0%).

**Conclusion:**

We suggest that to effectively mitigate the high prevalence of obesity among less-educated women, it may be necessary to help low-educated women who do not feel stressful develop strategies to combat their higher risk of obesity. We also suggest the need to conduct decomposition studies in countries which show significant relationships between education and obesity among women, and to create targeted policies to reduce this population’s overall risk of obesity.

## Background

Income and education, two representative indicators of socioeconomic status, have long been recognized as important determinants of obesity [1–3]. However, the relationship between income and obesity may not be the same as the relationship between education and obesity, and each of these relationships may manifest differently in men and women [1, 2]. Although some studies have yielded mixed results regarding the association between income and obesity in women [4, 5], education has been shown to have a strong negative association with obesity in studies conducted in the U.S. [6, 7], the U.K. [8], Sweden [9–11], France [12], China [13], Thailand [14], Singapore [15], Brazil [16], and South Korea [17–19].

Examining these results, we the authors became curious about the factors explaining the relationship between education and obesity among women. More precisely, which factors differentiating less-educated women from highly-educated women put less-educated women at a higher risk of obesity? Do these differences arise from the composition of socioeconomic characteristics (that is, a difference in observed characteristics between the two groups), or is it because less-educated women with a given set of characteristics are much more likely to be obese than highly-educated women with the same characteristics (that is, a difference in the estimated coefficient of the characteristics between the two groups)?

From an academic viewpoint, it may be important to identify which factors account for the relationship between education and obesity in women in order to enable researchers to develop and test new theories. Doing so would also help policy-makers to design and implement effective policies to combat obesity in less-educated women. For example, if the individual contribution of a given factor to the relationship between education and obesity in women is positive, large, and significant, we may expect that an increase in the magnitude of this factor over time may increase the disparity that we presently see between rates of obesity in less- and highly-educated women, and that if policy-makers effectively decrease the magnitude of this factor, we might see a reduction in this disparity. Despite the importance of these factors, to the best of our knowledge, no attempts have yet been made to explore the factors explaining different rates of obesity among differently-educated women in a detailed, rigorous way.

This study therefore estimated the overall and separate contributions of sets of factors explaining the different rates of obesity among women with different levels of education using a decomposition approach, extended from the Oaxaca-Blinder method [20, 21]. We hypothesized that factors explaining the relationship between education and obesity in women might be decomposed into 1) a portion attributable to the differences in observed characteristics between the two education groups, and 2) a portion that is explained by differences in the estimated coefficients between the two education groups. We also postulated that there might be dominant factors which have more power to explain the relationship between education and obesity in women. We chose to analyze datasets from national surveys in South Korea because this relationship is particularly strong in South Korea, where obesity rates are more than twice as high among women with a high school education or less than they are among women with a college education or higher. Furthermore, studies have shown that South Korean women put more effort into losing weight than women in 22 countries [22], so we studied the situation of South Korean to determine whether highly-educated women make greater efforts than less-educated women to avoid weight gain or to lose weight.

## Methods

### Datasets and study sample

This study used 5 years’ worth of data from the Korea National Health and Nutrition Examination Surveys (KNHANES, 2010–2014). These surveys are conducted annually by the Korean Centers for Disease Control and Prevention, and solicit data from the general, non-institutionalized population through a stratified, multistage probability sampling design. These surveys are nationally representative datasets, containing abundant information on South Koreans’ demographics, socioeconomic status, health, and lifestyles. Of the 41,102 individuals in the 5 years of data, 22,456 were women. 16,877 of these were women aged 25 years or above who had most likely completed their formal education [23]. Of these, we selected 16,391 women after excluding 486 women who were pregnant or breastfeeding at the interview date, for either status is likely to affect body weight. We then used 14,577 women participants with complete information as a study sample (88.9% of the total), because there was no evidence of statistical differences regarding important demographic characteristics between groups with and without complete information (*p*-value = 0.187 for age and 0.275 for residential area). All KNHANES participants provided written consent to participate in the survey and for their personal data to be used. The data we used are publicly available, and the institutional review board of our organization provided ethical approval for our study.

### Measures and variables

First, we calculated each participant’s body mass index (BMI) based on their height and body weight, which were measured through physical examinations and available from the KNHANES. In accordance with the guidelines proposed by the World Health Organization (WHO), and considering that Asians generally have lower BMIs on average [24], we defined obesity as a body mass index of 25 or higher and modelled our dependent variable to equal one or zero according to whether a participant is obese or not, respectively.

Next, we measured participants’ education level. We defined a participant’s education level as the highest level of formal education they had completed as of the interview date. We then divided participants’ education level into two categories: a high school education or less (≤ 12 years of education), and attaining or working on a college degree or higher (≥ 13 years of education). Based on these categories, we divided our participants into a less-educated and a highly-educated group.

Independent variables include: age (25–34, 35–44, 45–54, 55–64, or 65 years and older); marital status (married or non-married, where non-married included never married, separated, widowed, or divorced); residential area (urban or rural); occupational status (employed or not employed, where not employed included those who had no paid work); household income (below median income, or median income or higher, where income was adjusted for household size by the square-root equivalence scale and median income was as defined by all participants’ information) [25]; current smoking status (smoking or non-smoking); risk of alcohol intake (no or low, or medium or higher, according to WHO’s sex-specific guidelines for risk of acute problems from drinking) [26]; walking exercise activity (active or inactive, according to whether a woman walks for at least 30 min per day at least 5 days per week) [27]; and self-perceived stress level (stressed or not stressed).

### Statistical analyses

In this study, we performed a six-fold analysis. First, we applied *χ*^2^-tests to determine whether the distribution in participants’ characteristics differed between our two groups.

Second, we examined whether age modified or confounded the relationship between education and obesity in women [28]. Because age confounded the relationship in both the unadjusted and adjusted models, we included age as a confounder in the analysis without stratifying the analysis by age categories.

Third, we continued to re-categorize each of the characteristics and re-define each characteristic’s reference category differently until both strong multicollinearity and a lack of goodness-of-fit disappeared in each model, because the decomposition analyses are based on multivariate logistic regression models for each education group. As a result, we constructed final models whose variance inflation factor values were less than 2.4 and had *p*-values based on the Hosmer–Lemeshow statistic of 0.565 for the less-educated group and 0.790 for the highly-educated group.

Fourth, we estimated the predicted prevalence (%) of obesity (PPO) (and its 95% confidence intervals, or CIs) of participants for each characteristic, where participants’ PPO denotes the average value of predicted probabilities that each participant would be obese when she belongs to a specific category of a characteristic but her other characteristics remain the same. The PPO estimates helped us to compare 1) the adjusted prevalence of obesity of participants across different categories of each characteristic in the same education group, and 2) the adjusted prevalence of obesity of participants belonging to a specific category of a given characteristic between two education groups.

Fifth, in order to decompose the difference in obesity rates between the two groups and discern characteristics’ separate contributions to the relationship between education and obesity, this study used an extended Oaxaca-Blinder decomposition method [29–31]. Following this method, we estimated the separate contribution of a certain observed characteristic (like the high proportion of women aged 25–34 years in our study) to the relationship between education and obesity by assigning these characteristics a percentage value. We then summed up all of the separate contributions to obtain “the contribution of overall composition effects.”

In addition, we noted that the association of a certain observed characteristic with obesity in women was sometimes different between our two groups, as suggested by the difference in the estimated coefficient of the characteristic between the two groups, which may account for the difference in obesity rates between the two groups. Therefore, we estimated the separate contribution of the differences in the association with being obese by education to the difference in obesity prevalence in the two education groups. We then summed over all such separate contributions to obtain “the contribution of pure association effects.”

In addition, we estimated the separate contributions of differences in the constant term coefficients between the multivariate logistic regression models for less- and highly-educated women to the difference in obesity rates between the two groups, which we call “the contribution of the group-specific effect.” Indeed, the contribution of the group-specific effect represents a contribution to the difference in obesity rates between the two groups that cannot be accounted for by all independent variables in each model under investigation, neither through any observed characteristic nor through its estimated coefficient. We then combined “the contribution of pure association effects” with “the contribution of the group-specific effect” and named it “the contribution of overall association effects.” To summarize, whereas the “overall composition effects” denote contributions due to the differences in observed characteristics between the two groups, the “overall association effects” denote the contributions due to the differences in the estimated coefficients and constant terms between the two groups when women’s obesity regressed in the observed characteristics of each group.

Finally, in order to explore changes in the contributions among models with different sets of independent variables, we constructed a hierarchy of three models and conducted three analyses. Model 1 uses demographic variables (age, marital status, and residential area) as independent variables. Model 2 uses socioeconomic variables (occupational status and household income) along with the independent variables used in Model 1. Model 3 uses lifestyle variables (smoking, risk from alcohol intake, walking exercise activity, and self-perceived stress) along with the independent variables used in Model 2.

We conducted all analyses with consideration for the complex survey design and set the statistical significance to an alpha level of 0.05. We used SAS 9.4 (SAS Institute, Cary, NC, USA) and STATA 15 software (StataCorp, College Station, TX, USA).

## Results

### Participant characteristics

Table 1 shows the distributions of study sample characteristics for both groups. The number of less-educated women (10,806, or 74.1% of the study’s total) were greater than that of highly-educated women (3771, or 25.9% of the study’s total). Obesity was nearly twice as prevalent among less-educated women (34.3%; 95% CI: 33.2–35.5%) as it was among highly-educated women (16.0%; 95% CI: 14.6–17.5%), with a very large difference (18.3 percentage points).

**Table 1:** Distribution (%) of study sample characteristics by education level among women: Korea National Health and Nutrition Examination Survey, 2010–2014 (*N* = 14,577)

Characteristics	Less-educated		Highly-educated	
	Proportion	(95% CI)	Proportion	(95% CI)
Obesity	34.3	(33.2-35.5)	16.0	(14.6-17.5)
***Demographic***				
Age, years				
25–34	8.8	(8.0-9.7)	41.3	(39.1-43.4)
35–44	17.6	(16.6-18.7)	36.6	(34.7-38.6)
45–54	26.6	(25.5-27.7)	16.5	(15.0-18.1)
55–64	21.5	(20.5-22.4)	4.5	(3.8-5.2)
≥ 65	25.6	(24.5-26.7)	1.2	(0.9-1.5)
Marital status				
Married	73.3	(72.1-74.4)	71.1	(69.0-73.1)
Non-married	26.7	(25.6-27.9)	28.9	(26.9-31.0)
Residential area				
Urban	76.7	(73.6-79.5)	90.7	(88.5-92.6)
Rural	23.3	(20.5-26.4)	9.3	(7.5-11.5)
***Socioeconomic***				
Occupational status				
Not employed	50.8	(49.5-52.1)	42.6	(40.6-44.6)
Employed	49.2	(47.9-50.5)	57.4	(55.4-59.4)
***Household income***				
Median or higher	43.4	(42.0-44.8)	73.0	(71.1-74.9)
Below median	56.6	(55.2-58.0)	27.0	(25.1-28.9)
***Lifestyle***				
Current smoking				
Non-smoking	92.8	(92.0-93.4)	95.4	(94.5-96.2)
Smoking	7.3	(6.6-8.0)	4.6	(3.8-5.5)
Risk from alcohol intake				
No or low	73.4	(72.3-74.5)	64.3	(62.3-66.3)
Medium or higher	26.6	(26.6-35.7)	35.7	(33.7-37.7)
Walking exercise				
Inactive	65.2	(64.0-66.4)	64.4	(62.5-66.3)
Active	34.8	(33.6-36.0)	35.6	(33.7-37.6)
Self-perceived stress				
Not stressed	73.0	(72.0-73.9)	69.7	(68.0-71.4)
Stressed	27.0	(26.1-28.0)	30.3	(28.6-32.0)
N	10,806		3771	

*Note*: *N* number, *Obesity* body mass index ≥25, *CI* confidence interval

Less-educated denoted high school education or less. Highly-educated denoted college degree or higher

All analyses were conducted considering the complex survey design

Non-married includes never-married, separated, widowed and divorced

Not employed included participants who had no paid work

Income was based on equivalized household income at each survey year

In our study, less-educated women were proportionally older (i.e., more of them were aged 45 years or older) than highly-educated women. Furthermore, more women in this first group were married, residents of rural areas, unemployed, had lower household incomes than the median value, smokers, exposed to no or low risk of alcohol intake, and perceived themselves as not stressed compared to our group of highly-educated women. Participants’ characteristics differed significantly in their distributions between the two groups except for marital status (*p* = 0.072) and walking exercise (*p* = 0.468).

### Predicted prevalence of obesity

Table 2 shows the PPO for women belonging to a given category of a characteristic across both groups, adjusted for the other characteristics.

**Table 2:** The predicted prevalence of obesity (%) for every category of a characteristic by education level among women: Korea National Health and Nutrition Examination Survey, 2010–2014 (*N* = 14,577)

Characteristics	Less-educated			Highly-educated		
	Rate	(95% CI)	*p*	Rate	(95% CI)	*p*
***Demographic***						
Age, years						
25–34	24.7	(20.7-28.7)	<.001	14.1	(11.5-16.6)	<.001
35–44	27.6	(24.9-30.2)	<.001	17.5	(14.8-20.2)	<.001
45–54	32.7	(30.5-34.9)	<.001	22.4	(18.2-26.7)	<.001
55–64	39.0	(36.7-41.3)	<.001	25.7	(18.8-32.6)	<.001
≥ 65	38.4	(36.0-40.8)	<.001	40.3	(27.9-52.7)	<.001
Marital status						
Married	33.1	(31.7-34.4)	<.001	24.7	(21.2-28.2)	<.001
Non-married	29.8	(27.6-32.1)	<.001	21.6	(16.9-26.3)	<.001
Residential area						
Urban	31.4	(30.0-32.7)	<.001	23.1	(19.9-26.2)	<.001
Rural	35.6	(33.3-37.8)	<.001	26.2	(20.0-32.4)	<.001
***Socioeconomic***						
Occupational status						
Not employed	32.9	(31.3-34.6)	<.001	23.4	(20.0-26.9)	<.001
Employed	31.5	(29.9-33.1)	<.001	24.1	(20.2-28.0)	<.001
Household income						
Median or higher	30.2	(28.5-31.9)	<.001	20.0	(16.9-23.1)	<.001
Below median	34.3	(32.6-36.0)	<.001	27.3	(22.9-31.7)	<.001
***Lifestyle***						
Current smoking			<.001			<.001
Non-smoking	32.4	(31.1-33.6)	<.001	23.3	(20.2-26.4)	<.001
Smoking	29.3	(24.8-33.8)	<.001	30.3	(20.1-40.5)	<.001
Risk from alcohol intake						
No or low	30.7	(29.4-32.1)	<.001	23.1	(19.9-26.3)	<.001
Medium or higher	36.0	(33.6-38.4)	<.001	25.7	(21.0-30.4)	<.001
Walking exercise						
Inactive	32.6	(31.2-34.0)	<.001	22.7	(19.4-26.0)	<.001
Active	31.5	(29.6-33.4)	<.001	25.7	(21.4-30.0)	<.001
Self-perceived stress						
Not stressed	32.0	(30.8-33.2)	<.001	23.0	(19.8-26.2)	<.001
Stressed	35.4	(30.6-40.3)	<.001	37.1	(27.5-46.7)	<.001
N	10,806				3771	

*Note*: *N* number, *Obesity* body mass index ≥25, *CI* confidence interval

Less-educated denoted high school education or less. Highly-educated denoted college degree or higher

All analyses were conducted considering the complex survey design

Non-married includes never-married, separated, widowed and divorced

Not employed included participants who had no paid work

Income was based on equivalized household income at each survey year

Compared to the highly-educated women, less-educated women in this study showed a higher PPO in most categories. In particular, their PPOs were higher by 10 or more percentage points in women who were aged 25–34 years (24.7%; 95% CI: 20.7–28.7%), 35–44 years (27.6%; 95% CI: 24.9–30.2%), 45–54 years (32.7%; 95% CI: 30.5–34.9%), 55–64 years (39.0%; 95% CI: 36.7–41.3%), had a household income at the median value or higher (30.2%; 95% CI: 28.5–31.9%), or exposed to a medium or high risk of alcohol intake (36.0%; 95% CI: 33.6–38.4%). By contrast, less-educated women had a lower PPO in three categories: women aged 65 years or more (38.4%; 95% CI: 36.0–40.8%), smokers (29.3%; 95% CI: 24.8–33.8%), and self-perceptions of stress (35.4%; 95% CI: 30.6–40.3%).

### Contributions of each demographic, socioeconomic, and lifestyle variable by group

Table 3 shows the outcomes of decomposition analyses of the differences in obesity prevalence between the low- and high-educated women’s groups. In Model 1, which only uses demographic variables as independent variables, the overall composition effects accounted for 36.4% of the difference in obesity rates between the two groups, and the overall association effects accounted for 63.6% of the difference.

**Table 3:** Contributions (%) due to composition and association effects to the difference in obesity rates between less- and highly-educated women for each model: Korea National Health and Nutrition Examination Survey, 2010–2014 (*N* = 14,577)

Characteristics	Less-educated vs. Highly-educated			
	Difference	Model 1	Model 2	Model 3
**Difference in obesity prevalence**	18.3			
**Overall composition effects**		36.4	41.8	44.2
Demographic		36.4	33.5	38.2
Socioeconomic		–	8.3	8.3
Lifestyle		–	–	−2.3
**Overall association effects**		63.6	58.2	55.8
Demographic		13.0	11.5	9.8
Socioeconomic		–	3.5	3.0
Lifestyle		–	–	43.6
Group-specific		50.6	43.2	−0.6
**Total**		100.0	100.0	100.0

*Note*: *N* Number

Less-educated denoted high school education or less. Highly-educated denoted college degree or higher

Model 1 included demographic variables (age, marital status, and residential area) as independent variables

Model 2 added socioeconomic variables (occupational status and household income) to the independent variables in Model 1

Model 3 added lifestyle variables (smoking, risk from alcohol intake, walking exercise activity, and self-perceived stress) to the independent variables in Model 2

In Model 2, in which socioeconomic variables were added to the independent variables listed in Model 1, the contribution of the overall composition effects increased to 41.8% (an increase of 5.4 percentage points), whereas the contribution of the overall association effects decreased to 58.2%. In Model 3, where lifestyle variables were added to the independent variables of Model 2, the overall composition effects made a larger contribution than in Model 2 (increasing 2.4 percentage points to 44.2%), and showed a concomitant decrease in the contribution of the overall association effects to 55.8%. In particular, the group-specific effect made a very large contribution, as much as 50.6%, in Model 1, but its contribution dropped to 43.2% in Model 2 and then almost disappeared in Model 3 (− 0.6%, *p*-value = 0.972). This implies that Model 3 is well-suited to explain the difference in obesity rates between these two groups, yielding a trivial contribution by the part that cannot be accounted for through the model.

### Individual contributions by significant characteristics

**Fig. 1:**
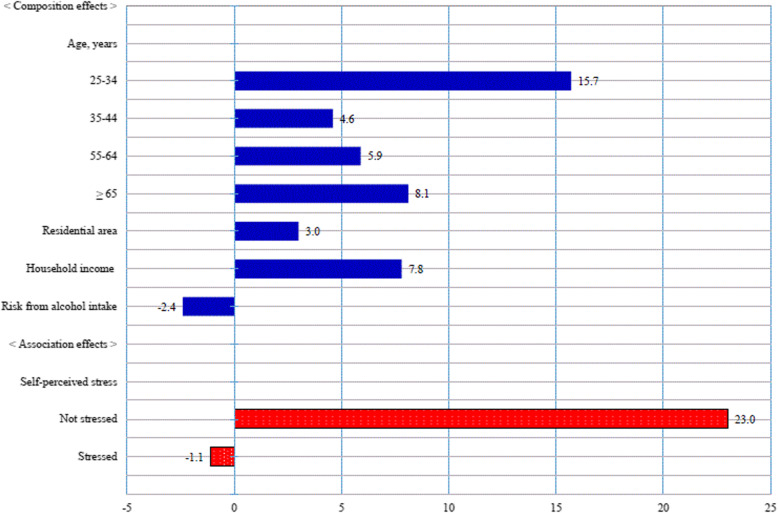
Individual contributions (%) of characteristics whose composition or association effect was statistically significant to the difference in obesity rates between less- and highly-educated women: The Korea National Health and Nutrition Examination Survey, South Korea, 2010–2014 (*N* = 14,577)

Figure 1 shows the individual contributions of those characteristics whose composition or association effect was statistically significant at a *p*-value < 0.05 to the difference in obesity rates between our two groups in Model 3.

As for the composition effect, differences in the distributions of women’s age categories between the two groups – for example, aged 25–34 years (15.7%), aged 65 years or more (8.1%), aged 55–64 years (5.9%), and aged 35–44 years (4.6%) – made major positive contributions, as did women’s household income (7.8%) and residential area (3.0%). In contrast, the difference in the distributions of women at risk from alcohol intake between the two groups contributed negatively to the difference in obesity rates between the two groups (− 2.4%).

Regarding the association effect, the characteristic explaining the difference in obesity rates between the two groups was self-perceived stress. In other words, the difference between the degree to which women in each group reported that they did not feel stressful made a large positive contribution to the difference in obesity rates between the two groups (23.0%), in sharp contrast to the negative contribution (− 1.1%) of the difference between the two groups for women who reported that they felt stressed.

## Discussion

Although education and income are both known to determine peoples’ risk of obesity, it seems very important to investigate the relationship between education and obesity and the relationship between income and obesity separately, because education may be attained earlier in life and determine peoples’ income later in life [1, 2, 32]. Previous studies have reported an inverse association between education and obesity among women, both in developed countries [6–12, 17, 19] and developing countries [13–16, 33], except for a few studies [15, 34, 35]. In line with these findings, our study found that obesity was more than twice as prevalent among less-educated women (34.3%) than among highly-educated women (16.0%) in South Korea.

In addition, according to results obtained from our study of an extended Oaxaca-Blinder decomposition method (Model 3 in Table 3), in explaining the differences in obesity rates between the two groups, the overall association effects were much higher than the overall composition effects between the two groups (55.8% vs. 44.2%).

### Association effects

Of the association effects, lifestyle variables turned out to be the most important contributor (43.6%) to the difference in obesity rates between the two groups (Model 3 in Table 3). This means that the relationship between women’s lifestyle variables and obesity differs greatly between our two groups. One possible explanation for this is that through change in lifestyle behaviors, high-educated women may control their body weight to a greater extent than low-educated women, as shown in a British study [8].

Because no study is comparable to our study in terms of the results obtained from decomposition analyses, we compare our study to studies which investigate the effects of lifestyle behaviors on the associations between education and obesity [9, 35, 36]. Some studies have shown that smoking influences the association between education and obesity among Australian women [36] and that heavy alcohol use significantly influenced the association between education and obesity among Swedish women [35]. Meanwhile, a recent study noted that education level may modify the association between lifestyle behaviors and obesity in South Korea [37].

Indeed, as for psychosocial stress, very little is known about the relationship between education, psychological stress, and obesity even, in developed countries. However, our decomposition analysis found that through its association effect, self-perceived stress was the most important characteristic explaining the relationship between education and obesity among women. Although its methods are not comparable to our study, a study of middle-aged Swedish women showed that psychosocial stress, reproductive history, and unhealthy dietary habits explained a large portion of the association of low socioeconomic status with obesity [9]. In particular, our decomposition results, together with the PPO results displayed in Table 2, led us to two intriguing conclusions.

First, our decomposition study showed that regardless of their education, women who feel stressed are more likely to be obese than women who do not feel stressed. Our PPO results were consistent with this finding: they indicated that the PPO of less-educated women who felt stressed (35.4%) was higher than those who did not (32.0%) – we found similar results when measuring the relationship between PPO and stress in highly-educated women (37.1% vs. 23.0%). This may be supported by the results of studies from Sweden [38], the U.S. [39], and South Korea [19] – i.e., that stress is known to be associated with a higher BMI in women. However, these studies did not conduct analyses stratified by women’s education.

Second, our decomposition study provided a noteworthy result: not feeling stressed made a large positive contribution to the relationship between education and obesity through association effects (23.0%), and feeling stressed made a small negative contribution to the relationship between education and obesity through the association effects (− 1.1%). This result suggests that a large portion of the relationship between education and obesity in women might arise because, among the women who do not feel stressful, less-educated women are much more likely to be obese than highly-educated women. Our PPO results seem to support this in the sense that among women who did not feel stressful, less-educated women were much more likely to be obese (PPO 32.0%) than highly-educated women (PPO 23.0%).

Following these results, we may ask ourselves why, among women who do not feel stressed, less-educated South Korean women are more likely to be obese than highly-educated women. One plausible reason is that highly-educated women in South Korea control their weight more effectively because they may have better knowledge and access to resources regarding their health (e.g., the significance of exercise and caloric intake) [40, 41]. Another plausible explanation might be that South Korean women often feel that, to become or remain employed, they must adhere to the expectations of employers and coworkers in a male-dominated workforce. Therefore, highly-educated women may be more motivated to accept this social pressure to be thin and, further, be better-equipped to meet social norms, possibly due to their class upbringing [5, 42, 43].

One study reported that women in the Asia-Pacific region feel more overweight and put more effort into losing weight than women in four other regions around the world [22]. A high proportion of South Korean women, in particular, indicated that they were trying hard to lose weight (77%), which suggests that South Korean society has internalized that idea that women’s social value is tied to their thinness [17].

### Composition effects

As for the composition effects, the educational difference in demographic variables was a major contributor (38.2%) to the educational difference in obesity rates, and the difference in socioeconomic variables was of secondary importance (8.3%).

When we estimated the separate contributions of each variable, educational differences in the distributions of age categories turned out to make a large contribution to the relationship between education and obesity. For example, 15.7% of the contribution was due to educational differences in the distribution of the category of 25–34 years of age; by contrast, educational differences in the distribution of the category of 65 years or older of age contributed 8.1%. These results cannot be compared with those of other studies because no previous study has explored the role of age in the composition effect of the relationship between education and obesity. Instead, some studies of middle-aged and working-age populations have shown that less-educated women are more likely to be obese than highly-educated women [9, 11, 44].

Among socioeconomic variables, educational differences in the distribution of women’s household income made a positive contribution (7.8%) to explain the relationship between education and obesity in women. In light of a lack of relevant previous studies, we instead reviewed studies which tackled the association between household income and obesity. These studies showed mixed results. For example, household income was negatively associated with obesity among women in European countries and Brazil [45, 46], exhibited no significant correlation with obesity among women in several Asian countries, including China, Thailand, and the Philippines [32, 47], and was positively correlated with obesity among wealthier Korean and Indian women [18, 48, 49]. Researchers need to consider the possibility that education affects both income and obesity because, all other things being equal, highly-educated women are more likely to have higher incomes [1, 50] and control their body weight [22], as implied by a study conducted in South Korea [28].

According to our decomposition analysis, the relationship between education and women’s residential areas made a positive contribution (3.0%) to the relationship between education and obesity among women. Despite a lack of similar or relevant decomposition studies which explain the separate contributions of education and residential area to the relationship between education and obesity among women, previous studies have produced mixed results concerning the effect of a residential area on this relationship. A study conducted in Mexico found a positive association between education and obesity among women in rural areas [51]. Comparably, a study of Peruvian women [52] found no evidence of any association between education and obesity in rural areas, but showed that high levels of education had a negative association with obesity in urban areas. This seems consistent with findings that highly-educated women in Brazil are less likely to be obese, and this effect was stronger in more urbanized regions [46].

### Strengths and limitations of this study

The present study assessed data from a nationally representative sample of adult women, which provided abundant information on anthropometric measures as well as demographic, socioeconomic, and lifestyle characteristics. To the best of our knowledge, this study is the first to employ an extended Oaxaca-Blinder decomposition method to explore the factors contributing to the relationship between education and obesity in women and quantify their separate contributions (in terms of both composition and association effects).

This study has several limitations. First, it precludes any definitive expression of the causal relationship between education, obesity, and other individual characteristics. Second, given the lack of related information, this study did not consider the quality of women’s education. Third, according to results of an additional analysis regarding whether independent variables were effect modifiers in the relationship between education and obesity in women, we found that self-perceived stress was potentially an effect modifier. Therefore, future studies in this vein will need to stratify their analyses by education level as well as by self-perceived stress. Fourth, although it is not unobserved, the differences in time preference among women may have affected some of their characteristics, including education level and obesity status [53–55]. Moreover, we did not use the information on aerobic and muscle-strengthening physical activities and dietary intake to construct additional independent variables for two reasons. The first was to avoid a reverse causality bias, because no information was given whether participants aimed to control or manage their body weights through such physical activities or diet. The second was the fact that many participants did not respond to questions related to such information, and some questions were not surveyed for one or 2 years during our study period; consequently, numerous participants may have dropped out of the analysis.

## Conclusions

At present, no rigorous study has investigated the factors explaining the relationship between education and obesity in women. Using an extended Blinder-Oaxaca decomposition method, we found that different rates of obesity among South Korean women with different levels of education was mainly due to association effects rather than composition effects, and was due to differences in the association of women’s lifestyle characteristics with obesity in particular. We also found that self-perceptions of stress played a large part in this relationship. From a policy perspective, therefore, this study emphasizes the importance of developing an enhanced governmental education policy which focuses on reducing less-educated women’s risk of obesity.

When researching the relationship between education and obesity in women, researchers need to pay attention to the association effects of lifestyle characteristics, including self-perceptions of stress. In addition, it appears necessary to examine whether these results are valid in other socio-economic settings. A more precise understanding of the factors contributing to educational differences in obesity prevalence among women may help policy-makers to establish tailored policies and more efficiently allocate resources to reduce these differences.

## Data Availability

Detailed information on the survey design and characteristics are provided at http://knhanes.cdc.go.kr, and the KNHANES data is publicly avilable from the link.

## Abbreviations

KNHANES: Korea National Health and Nutrition Examination Surveys
PPO: Predicted prevalence of obesity
N: Number
CI: Confidence interval
